# Ultrasound pulse repetition frequency preferentially activates different neuron populations independent of cell type

**DOI:** 10.1088/1741-2552/ad731c

**Published:** 2024-09-09

**Authors:** Jack Sherman, Emma Bortz, Erynne San Antonio, Hua-an Tseng, Laura Raiff, Xue Han

**Affiliations:** 1 Department of Biomedical Engineering, Boston University, Boston, MA, United States of America; 2 Department of Pharmacology and Experimental Therapeutics, Boston University, Boston, MA, United States of America

**Keywords:** transcranial ultrasound, neuromodulation, GCaMP7f, mechanosensitive ion channels, parvalbumin interneurons, excitatory neurons, resonant frequencies

## Abstract

*Objective*. Transcranial ultrasound (US) stimulation serves as an external input to a neuron, and thus the evoked response relies on neurons’ intrinsic properties. Neural activity is limited to a couple hundred hertz and often exhibits preference to input frequencies. Accordingly, US pulsed at specific physiologic pulse repetition frequencies (PRFs) may selectively engage neurons with the corresponding input frequency preference. However, most US parametric studies examine the effects of supraphysiologic PRFs. It remains unclear whether pulsing US at different physiologic PRFs could activate distinct neurons in the awake mammalian brain. *Approach*. We recorded cellular calcium responses of individual motor cortex neurons to US pulsed at PRFs of 10, 40, and 140 Hz in awake mice. We compared the evoked responses across these PRFs in the same neurons. To further understand the cell-type dependent effects, we categorized the recorded neurons as parvalbumin positive fast spiking interneurons or putative excitatory neurons and analyzed single-cell mechanosensitive channel expression in mice and humans using the Allen Brain Institute’s RNA-sequencing databases. *Main results*. We discovered that many neurons were preferentially activated by only one PRF and different PRFs selectively engaged distinct neuronal populations. US-evoked cellular calcium responses exhibited the same characteristics as those naturally occurring during spiking, suggesting that US increases intrinsic neuronal activity. Furthermore, evoked responses were similar between fast-spiking inhibitory neurons and putative excitatory neurons. Thus, variation in individual neuron’s cellular properties dominates US-evoked response heterogeneity, consistent with our observed cell-type independent expression patterns of mechanosensitive channels across individual neurons in mice and humans. Finally, US transiently increased network synchrony without producing prolonged over-synchronization that could be detrimental to neural circuit functions. *Significance*. These results highlight the feasibility of activating distinct neuronal subgroups by varying PRF and the potential to improve neuromodulation effects by combining physiologic PRFs.

## 1. Introduction

Transcranial low-intensity ultrasound (US) neuromodulation represents a promising therapeutic strategy for neurological and psychiatric disorders [1, 2]. Noninvasive US neuromodulation typically uses lower fundamental frequencies of hundreds of kilohertz to a couple megahertz due to superior skull penetration [3–8]. In addition to fundamental frequency and acoustic pressure, various permutations of US pulse repetition frequencies (PRFs), durations, and intervals create an essentially infinite parameter space. As a mechanical energy wave, US interacts with cellular membranes through various acoustic phenomena which could induce phospholipid reconfiguration, alterations in membrane fluidity and permeability [9–13], or changes in membrane curvature [10]. While the exact mechanism by which US alters individual neurons is actively debated, the outcome of US neuromodulation ultimately depends on evoked neural activity changes that rely on intrinsic cellular biophysical and biochemical properties.

Though the exact physical interactions between US and cellular membranes remain unknown, mechanosensitive channels are generally thought to be critical in mediating cellular signaling changes during US neuromodulation [14–19]. Many mechanosensitive channels are widely expressed in the brain [20], such as the two-pore potassium K2P family [20–22], hyperosmolality-gated calcium-permeable TMEM63 family [20, 23], mechanosensitive cation channel Piezo family [20, 24, 25], and the transient receptor potential TRP families [19, 20, 26–30]. Among the large number of mechanosensitive channels, several members of the K2P, TRP and Piezo1 families have been implicated in US neuromodulation [19, 24–26, 31, 32] (supplemental table 1). For example, genetic knockout of Piezo1 [25] or knockdown of Trpm2 [26] reduced US-mediated neural and behavioral responses in mice. Similarly, genetic knockdown of TRPC1, TRPP1, and TRPP2 attenuated cellular calcium responses in cultured neurons *in vitro* [19].

Mechanosensitive channels are broadly distributed in brain tissue. However, it is unclear how mechanosensitive channel expression differs between individual cells in the brain. In this study, we first analyzed Allen Brain Institute’s single cell sequencing database and confirmed extensive variation of mechanosensitive channel expression among individual neurons in both mice and humans. Interestingly, some channels show stronger expression levels in excitatory neurons than inhibitory neurons, though the differences between cell types are much less pronounced than between individual neurons. Nonetheless, systemic variation among cell types could result in distinct mechano-sensitivity and thus differential responses between cell populations to US.

The effect of US not only depends on the direct activation of individual neurons but also on subsequent network responses in intact brain circuits. At a fundamental frequency of around 0.5 MHz, typically used for transcranial neuromodulation, a beam from a single element US transducer can be focused to a cross-section of several millimeters in diameter and 10–20 mm in focal length. However, this still covers a large tissue volume, generally containing millions or more neurons and many neuronal fibers depending on the targeted brain region. This lack of focus at lower US fundamental frequencies is particularly prominent when studying US mechanisms in mouse models with a brain size of about 13 ×11 ×8 mm^3^, where around a quarter or more of the brain is sonicated. Indeed, experimental studies showed that US could produce both excitatory and inhibitory neural circuit changes in humans [3–7, 33] and animal models [25, 26, 34–37]. For example, in humans, 0.5 MHz US delivered at 1 kHz PRF suppressed somatosensory evoked potentials [33], whereas 0.25 MHz US at 500 Hz PRF excited the somatosensory cortex [5]. Our previous study also confirmed that US evoked more prominent cellular calcium responses in the motor cortex than the hippocampus in awake mice [35]. Despite the difficulty of dissociating direct US-evoked cellular effects from indirect network responses, US at 900 Hz PRF was recently shown to activate parvalbumin expressing interneurons at a population level, while suppressing the excitatory neuron population in the mouse hippocampus [34]. Similarly, US pulsed at 30 Hz, 300 Hz, and 1.5 kHz PRFs produced slightly different responses in excitatory versus inhibitory neuron populations [37].

Some mechanosensitive channels, such as TRPs and Piezo1, are permeable to cations including calcium, and thus their activation depolarizes the plasma membrane and increases intracellular calcium [19, 24–26]. Both membrane depolarization and intracellular calcium rise could engage downstream signaling that leads to increased spiking probability. Since US neuromodulation generally exhibits weak effects, the intracellular pathways downstream of mechanosensitive channel activation, in particular activation of voltage-gated sodium and potassium channels critical for action potential generation, likely provide additional amplification mechanisms to translate acoustic driven responses into spiking outputs [14–17, 19]. This is supported by the observation that US activation of mechanoreceptors TRPP and TRPC led to intracellular calcium increases, which subsequently recruit calcium-activated TRPM4 channels and voltage-gated T-type calcium channels to increase spiking probability in cultured neurons [19].

The kinetics of neuronal ion channels generally limit the speed of spiking and subthreshold membrane voltage fluctuations to a couple hundred hertz. Many neurons have intrinsic biophysical properties that render them more sensitive to stimulation at certain physiologic frequencies of a few hertz to tens of hertz. We recently demonstrated that when electrical stimulation frequency reaches 140 Hz, membrane voltage was poorly paced by individual electrical pulses [38]. Thus far, most studies have delivered US at PRFs on the order of kilohertz. However, it is plausible that pulsing US at physiologic frequencies could optimize the coupling of mechanosensitive channel activation to subsequent signaling pathways, which together could improve US modulation efficiency.

To examine whether US at different physiologic PRFs selectively influence neuronal response in the awake mammalian brain, we performed large-scale single cell calcium imaging from parvalbumin-positive interneurons and parvalbumin-negative predominantly excitatory neurons in awake head-fixed mice while pulsing 0.35 MHz US at 10 Hz, 40 Hz, or 140 Hz. We chose these frequencies based on previous observations that they likely engage biophysical signaling differently in motor cortex neurons. 10 Hz is within the beta frequency band, an intrinsically preferred frequency of the cortical-basal ganglia motor circuits, and readily supported by motor cortex neurons [39]. 40 Hz is within the gamma frequencies (∼30–100 Hz) that have been broadly linked to PV interneuron oscillations [40, 41]. Transcranial US pulsed at 40 Hz augmented gamma oscillations [42] and reduced amyloid beta in mouse models of Alzheimer’s disease [42, 43]. Furthermore, PV cells were implicated in the 40 Hz visual stimulation induced gamma oscillations and amyloid beta reduction [44]. 140 Hz is widely used in deep brain stimulation, which leads to better therapeutic outcomes than lower frequency stimulations. Our recent studies demonstrated that 140 Hz electrical stimulation robustly depolarized membrane voltage, scrambled spike timing and led to informational lesion [38].

Consistent with our analysis of the Allen Brain Institute’s single cell sequencing results showing prevalent and heterogeneous expression of many mechanosensitive channels across individual neurons, we detected diverse US-mediated cellular calcium responses across neurons, with many reliably activated. US-evoked calcium events in individual neurons exhibit the same characteristics as those naturally occurring during increased spiking probability, suggesting that US increases neuronal activity. Sham stimulation at these PRFs failed to activate neurons, ruling out possible activation by indirect auditory stimulation during US. By calculating pair-wise correlations between simultaneously recorded neurons, we confirmed that US transiently increased network synchrony without producing prolonged over-synchronization that may be detrimental to neurological functioning. Most intriguingly, by directly comparing the evoked responses to different PRFs in the same neuron, we revealed that most neurons were preferentially activated by a single PRF. As these PRFs have been shown to exert similar auditory nerve activation via cochlear fluid vibration [45], the observation that most activated neurons were responsive to only one PRF suggests that the observed effect cannot be explained by vestibular artifacts. Because of the frequency preference of individual cells, different populations of neurons were selectively recruited by specific PRFs. Finally, the evoked population responses across all PRFs tested were similar between the two neuron types, highlighting the importance of considering individual neuron heterogeneity regardless of conventionally defined cell type.

## 2. Results

### 2.1. Mechanosensitive channel expression is prominent and heterogeneous across individual neurons in the cortex and hippocampus of mice and humans

Mechanosensitive channels are critical in translating mechanical forces to cellular responses, and many members of the K2P, TRP, TMEM and Piezo families of mechanosensitive channels are widely expressed in the brain (supplemental table 1). However, there has been no systematic analysis of the expression patterns of these channels across brain regions or cell types. We first analyzed the Allen Mouse Brain Atlas *in-situ* hybridization gene mapping data to examine their brain-region dependent expression [46] (supplemental table 2, https://mouse.brain-map.org). We found that many mechanosensitive channels are widely expressed throughout the brain, and the expression levels for some channels are more variable than others. TRPC1, TRPM2&7, TMEM63B, and Piezo1 exhibit high expression levels throughout the brain, with some variations across brain regions. In contrast, Trpm3 expression is more restricted to the hippocampus, olfactory areas, and cerebellum. Such variation may underlie the reported difference in US mediated effects across brain regions [35].

**Figure 1.**
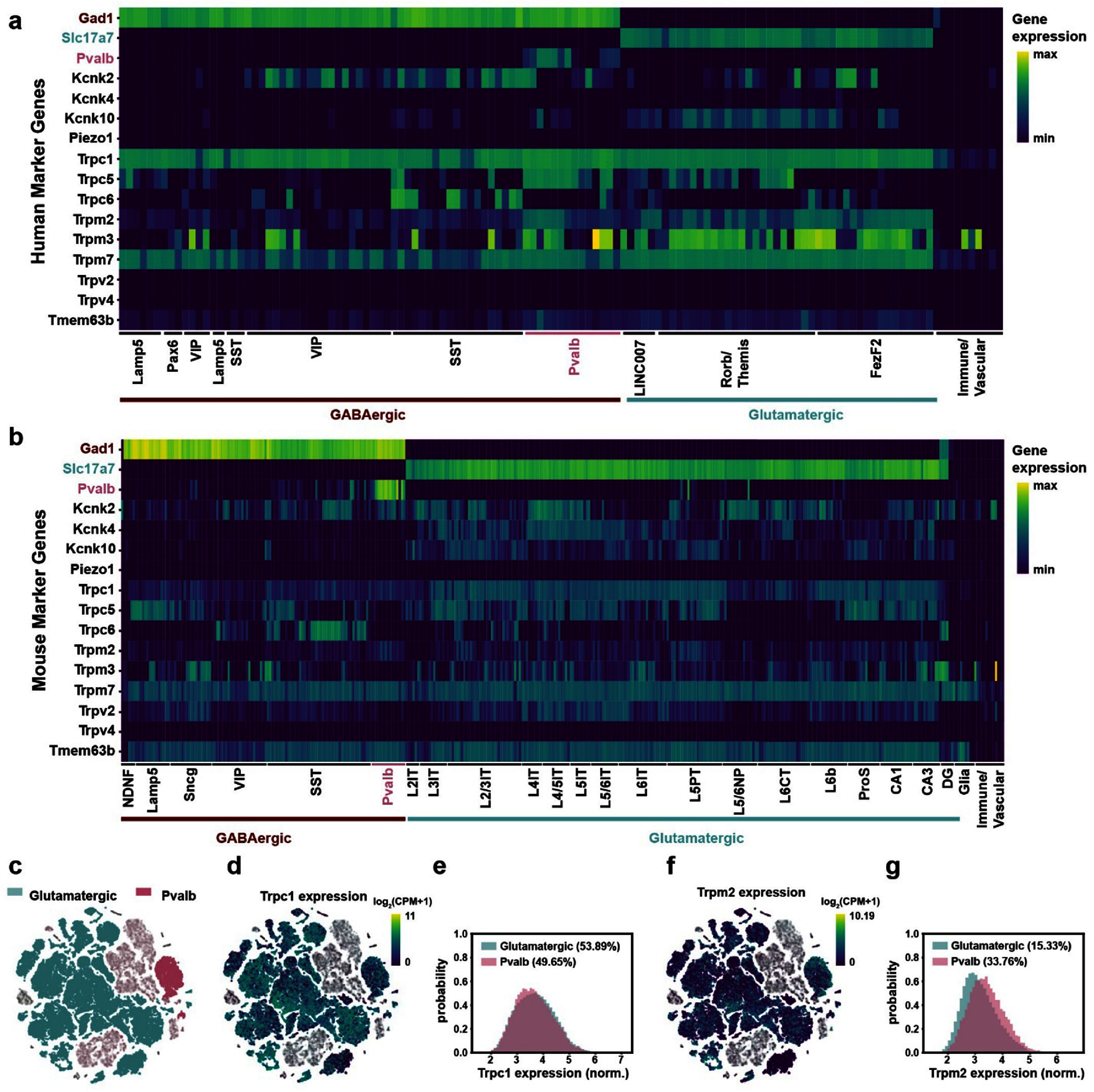
Widespread and heterogeneous expression of mechanosensitive channels in human and mice brains. (a) and (b) Heatmap of mechanosensitive ion channel gene expression in the human motor cortex (a) and the mouse motor cortex and hippocampus (b). Relative expression levels of example canonical marker genes and selected mechanosensitive channel genes are shown across common cell types within GABAergic and Glutamatergic neuron classes. Cell types are organized by hierarchical clustering. Gad1, inhibitory neuron marker gene; Slc17a7, excitatory neuron marker gene; and Pvalb, parvalbumin. Data are from Allen Institute for Brain Science, https://brain-map.org/atlases-and-data/rnaseq/human-m1-10x and https://brain-map.org/atlases-and-data/rnaseq/mouse-whole-cortex-and-hippocampus-10x. (c)–(f) Uniform Manifold Approximation and Projection (UMAP) representation of the mouse single cell sequencing results, colored by cell types ((c), glutamatergic, teal; Pvalb, pink; Other subtypes: gray), overlapped with Trpc1 expression (d) or Trpm2 expression (f). Expression was scaled by log_2_ (CPM +1), and the graphs were created with Cytosplore Viewer, https://viewer.cytosplore.org/. (e) and (g), Histograms of normalized expression of Trpc1 (e) and Trpm2 (g) in glutamatergic and PV neurons. Only cells with expression were included in probability density calculation, as shown by the percentage number in the legends. *N* = 30,461 PV cells and *N* = 976,358 glutamatergic cells.

To further evaluate single cell level expression of these mechanosensitive channels, we analyzed Allen Brain Institute’s single-cell RNA-sequencing transcriptome databases containing 76, 533 cells throughout the human motor cortex and 1.1 million cells throughout the mouse cortex and hippocampus [47–49]. We found that many channels are widely expressed across transcriptomically defined cell types in humans (figure 1(a)) and in mice (figure 1(b)). Interestingly, in humans, KCNK2 and TRPC6 appear to have stronger expression in inhibitory neurons, while KCNK10 are expressed predominantly in excitatory neurons (figure 1(a), supplemental table 3). Similarly, in mice, some channels, including TRAAK, TRPM3, TREK2, and TRPC1, have significantly higher expression levels in excitatory neurons (Slc17a7 positive) than inhibitory neurons (GAD1 positive) (figure 1(b), supplemental table 4). Curiously, Piezo1, previously demonstrated to play a role in mediating US modulation [24, 25], appeared negative in the transcriptome database, even though it is expressed across brain regions based on the *in-situ* hybridization gene mapping data (supplemental table 2). This may be due to Piezo1 expression being concentrated in a subset of neurons, as the transcriptome database uses the trimmed mean resulting a gene to be negative if it is expressed in <25% of a given cluster. Thus, we further analyzed Piezo1 expression in the more recent whole brain ABC atlas (https://knowledge.brain-map.org/abcatlas) and confirmed its expression in a small fraction of both excitatory and inhibitory neurons.

Previous studies also reported differences in US-evoked responses between PV positive fast-spiking interneurons and excitatory neurons [37]. Thus, we further compared gene expression patterns between PV positive interneurons and excitatory cells [47–49] (figures 1(c)–(g)). We observed a large variation in channel expression levels across individual neurons of both cell types, and the variation between individual cells was much more prominent than between cell types. For example, expression distributions of Trpc1 and Trpm2, both implicated in US neuromodulation [19, 26], showed small but significant variation between PV interneurons and glutamatergic excitatory cell populations (figures 1(e) and (g), Wilcoxon rank sum test, *p* < 0.01). Specifically, for Trpc1, a similar fraction (∼50%) of excitatory and PV cells had nonzero expression, and the expression level difference between the two populations was negligible ($\delta $ = −0.0363), indicating only a 3.63% probability that an excitatory neuron would have higher expression than a PV interneuron (figure 1(e)). Similarly, for Trpm2, 34% of PV cells showed expression, in contrast to 15% of excitatory cells, and the expression level across the PV population was only slightly higher than excitatory cells (figure 1(g), $\delta $ = 0.2315). Similar patterns are observed with the other channels examined, even though they are less explored in the context of US modulation (supplemental figure 1). Thus, the specific expression profiles of mechanosensitive channels in individual neurons are expected to have a greater impact on the outcome of US neuromodulation effect than canonical cell type.

### 2.2. Calcium imaging analysis of US stimulation effect on spiking-related calcium activity in individual cortical neurons in awake mice

While the exact mechanisms supporting the transformation of US acoustic pressure to changes in cellular activity remain elusive, US-evoked effect is ultimately dictated by individual neurons’ membrane biophysical properties and intracellular signaling [14, 15]. Most studies delivered US at high PRFs on the order of kilohertz, much higher than the maximum action potential rate neurons can support. Moreover, stimulation frequencies are also known to engage different neural activities. For example, in deep brain stimulation, pulse frequency is a critical consideration. We recently showed that 40 Hz electrical stimulation paced membrane potential and spike timing, but when electrical stimulation reached 140 Hz, spiking output failed to track stimulation pulses temporally [38]. To gain a deeper understanding of how neurons cellular properties influence US-evoked activities, we analyzed the neuronal response to US delivered at biophysically relevant frequencies.

**Figure 2.**
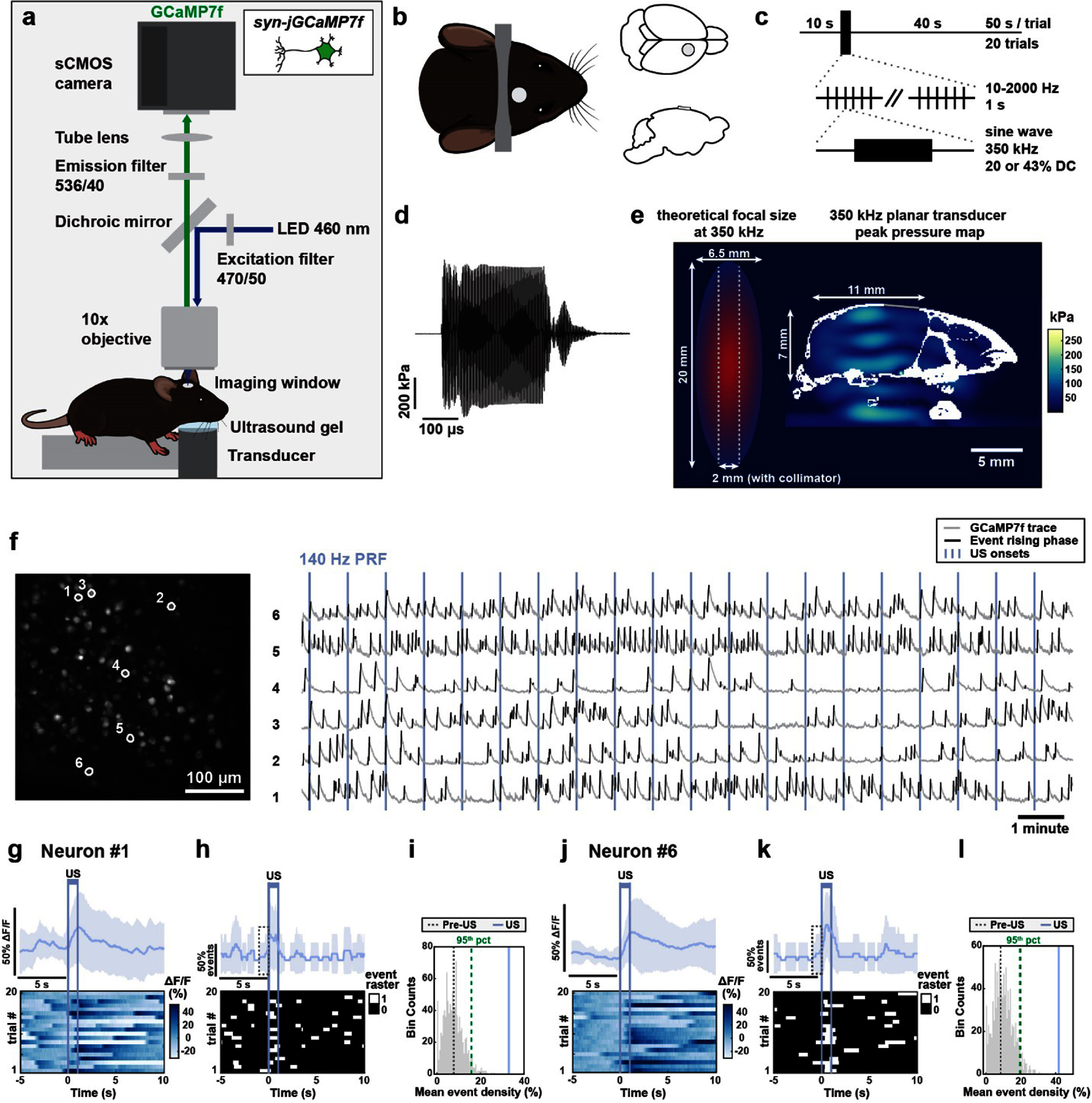
Cellular calcium imaging analysis of US-evoked calcium events associated with neuronal spiking in awake mice. (a) Experimental setup illustrating transcranial ultrasound stimulation on a head-fixed mouse during calcium imaging. The ultrasound transducer was placed beneath the mouse’s head and ultrasound gel acoustically coupled the transducer to the mouse. GCaMP7f expressing neurons were imaged with 460 nm LED excitation and 536/40 nm emission with an sCMOS camera. (b) Left: Imaging window and head bar placement, with the cranial window placed over the motor cortex. Right: Coronal and sagittal views of the cranial window. (c) Recording sessions consisted of 20 trials with 50 s each trial, containing a 10 s pre-US period followed by 1 s of 350 kHz US pulsed at 10, 40, 140, or 2000 Hz. (d) Example US waveform of the transducer recorded from an acoustic hydrophone in water. (e) Left: Theoretical −6 dB dimensions of 350 kHz US focal spot and 2 mm diameter beam collimator, to scale with mouse head dimensions for acoustic pressure simulation. Right: Simulation of peak acoustic pressure in the mouse brain during 350 kHz, 522 kPa US. Skull geometry is highlighted in white and glass imaging window in gray. Scale bar is 5 mm. (f) Left: An example field of view’s max-min fluorescence projection image with example neurons circled in white. Scale bar = 100 *µ*m. Right: GCaMP7f fluorescence traces from the six circled example neurons over 20 trials. GCaMP7f Δ*F*/*F* traces are in gray with detected calcium event rising phases highlighted in black. Vertical lines represent 140 Hz PRF US onset. (g), (j) Top: Trial-averaged Δ*F*/*F* aligned to US onset for neuron #1 (g) and neuron #6 (j). Shading corresponds to SD over 20 trials. Bottom: Normalized GCaMP7f Δ*F*/*F* heatmap across trials. Vertical purple lines depict US onset and offset. (h), (k), Top: Trial-averaged event density trace for neuron #1 (h) and neuron #6 (k). Shaded region represents SD over trials. Bottom: Binarized event rising phases over 20 trials. White represents timepoints with event rising phases and black represents timepoints with no event rising phases. Vertical lines represent US onset and offset. Dashed box depicts 1 s pre-US period. (i), (l) Illustration of statistical shuffling test used to determine neuron #1 (i) and neuron #6 (l) being modulated. Shuffled event density during non-US ‘baseline’ periods is shown in gray histogram, with the 95-percentile indicated by dashed green line. The observed event density during US is shown as a solid purple line, and the observed event density during the 1 s pre-US is shown as a dashed gray line.

We performed single cell calcium imaging in awake mice using GCaMP7f [50], an improved genetically encoded calcium sensor with higher sensitivity for detecting spike-related calcium transients (figure 2(a)). To target neurons specifically, we injected AAV9-syn-jGCaMP7f in the motor cortex of C57BL/6 mice, which drives gene expression in neurons via the synapsin (syn) promoter [51, 52]. We then placed a glass imaging cranial window above the pia for optical access of GCaMP7f labeled neurons (figure 2(b)). During each experiment, awake mice were head fixed under a custom wide-field imaging microscope with an US transducer placed under the chin (figure 2(a)) delivering 350 kHz planar US. Because of the small size of the mouse brain (13 mm × 14 mm × 8 mm), and a typical −6 dB focused US radiation volume around ∼6.5 mm × 6.5 mm × 20 mm at 350 kHz, we used a planar US transducer in this mechanistic study (figure 2(e)). We placed the planar transducer under the chin to accommodate for the optics above the head for high-resolution single cell imaging. While thin ring transducers have been applied in mouse imaging studies [34, 53], their focusing ability is rather limited and provides little advantage given the small size of the mouse brain. Similarly, collimators can laterally limit the US focal size to ∼2 mm depending on aperture size [54–56] (figure 2(e)); however, collimators cannot limit beam propagation axially and still sonicate a large fraction of the mouse brain. Further, collimators are too bulky to be integrated into our cellular optical imaging setup. Because of the limited spatial resolution of sub-megahertz US, in this study we focused on deriving a principled understanding of how different physiologic PRFs engage distinct neurons, which could guide the selection of US pulsing protocols during clinical translation, rather than testing the effect of sonicating a particular neural circuit related to certain behaviors or pathology.

US was pulsed at physiologically relevant frequencies of 10 Hz, 40 Hz, or 140 Hz and the traditionally used supra-physiological frequency of 2 kHz. We filled the space between the US transducer and the mouse’s chin with US gel to allow for efficient acoustic wave propagation into the brain. Sham conditions used the same preparation without US gel. To assess whether US-evoked calcium responses are consistent with intrinsic physiological calcium events associated with spiking, we pulsed US for 1 s every 50 s, allowing us to capture sufficient intrinsic calcium events during the intertrial intervals. We recorded a total of 20 trials, resulting in a duration of 16.7 min per session (figure 2(c)).

While the mouth and nasal cavities shape the acoustic pressure profiles, US nonetheless propagates through the tissue, bone and skin to the motor cortex regions imaged. To estimate the acoustic pressure intensity at the site of recording, we first recorded the spatial peak US pressure waveform in water with a needle hydrophone (figure 2(d)). The acoustic pressure waveform had a peak amplitude of 522 kPa, corresponding to *I*_SPPA_ of 9.11 W cm^−2^ in water (supplemental tables 5 and 6). We then used the recorded hydrophone pressure amplitude to computationally simulate the US pressure and intensity distributions in the mouse brain in k-Wave, as reported previously in Tseng *et al* [35] (figure 2(e), supplemental table 6). To approximate our experimental conditions and below-the-chin transducer placement, we modified the three-dimensional mouse skull from Chan *et al* [57] to include a motor cortex craniotomy, a glass imaging window, and air pockets above and below the tongue and above the soft palate. Our computational models revealed that stimulating with 522 kPa US resulted in an *in situ* spatial peak pressure of 289.5 kPa (supplemental table 6). At the site of calcium imaging, the simulated average peak pressure ranged 13–95 kPa, and the spatial peak pulse average intensity (ISPPA) ranged 0.2–3.16 W cm^−2^ (average intensity, 1.5 W cm^−2^). For computational model validation, we created a phantom mouse skull with the local bone structure above the motor cortex removed for hydrophone access. We then filled the phantom with US gel and measured pressure with a needle hydrophone at the approximate motor cortex imaging site. The measured pressure amplitude in the phantom mouse skull was ∼125 kPa, consistent with the upper bounds of our computational estimate.

To examine US effects on individual neurons, we extracted neuronal GCaMP7f fluorescence traces over time (figure 2(f)) and identified calcium events as those with large amplitude increases in GCaMP7f fluorescence (see methods). Across non-US periods, defined as the full recording duration excluding US periods and the five seconds immediately post-US, individual neurons had event rates of 3.10 ± 0.37 events/min (mean ± SD, *N* = 12 imaging sessions in 7 mice), consistent with the general observation using GCaMP7f [58]. While the rising phase of a calcium event is related to spiking, the falling phase is attributed to both cytosolic calcium clearance and GCaMP7f’s calcium ion dissociation kinetics. Thus, to analyze US effect on spike-related calcium activity, we binarized GCaMP7f fluorescence traces with ones depicting calcium event rising phases and zeros elsewhere (figures 2(g), (h), (j) and (k)) and computed calcium event density, a measure of the fraction of time a neuron has heightened spiking probability.

During each recording session, we noticed that many neurons increased event density during US (figures 2(g)–(l)). To determine whether a neuron was activated by US, we compared the mean calcium event density during US (1 s interval) across all 20 trials (figures 2(h) and (k)) to the shuffled baseline event density distribution (figures 2(i) and (l)). To generate the shuffled baseline distribution for each neuron, we averaged the event rate over 20 randomly selected 1 s intervals during ‘baseline’, defined as the full recording period excluding the 5 s period after US onset, and then repeated this procedure 1000 times. Neurons were deemed US-responsive if the event density during US was greater than the 95th percentile of the corresponding shuffled baseline distribution. Because calcium event rising phase in the recorded neurons lasted for 1.11 ± 0.67 s (mean ± SD, *N* = 177 646 events), events starting in the second before US may lead to false identification. Thus, we excluded neurons that had event density rates higher than the 95th percentile during the 1 s window prior to US.

### 2.3. US delivered at physiologically relevant PRFs reliably increased the rates of calcium events associated with natural neuronal spiking

**Figure 3.**
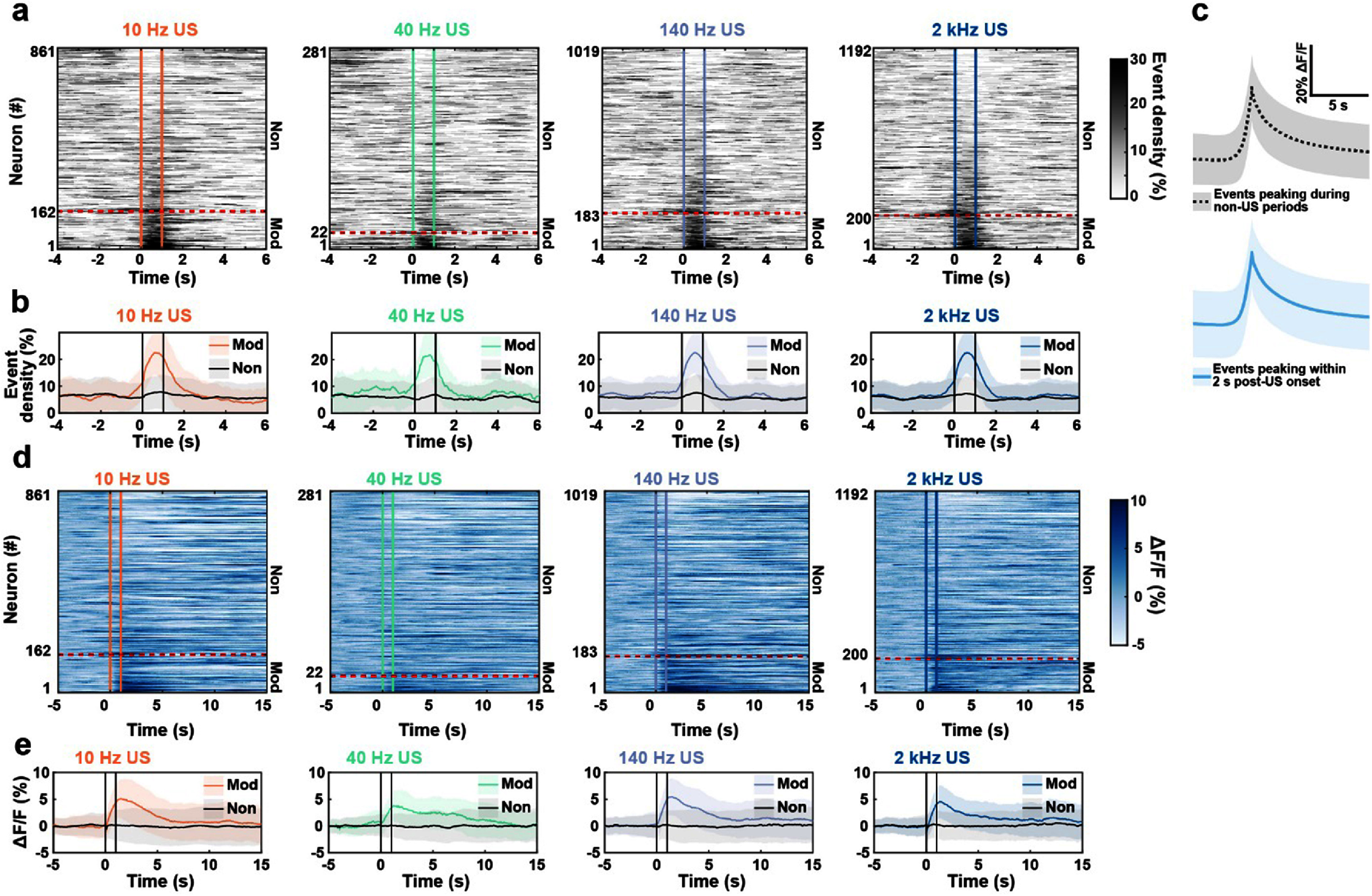
US delivered at 10 Hz, 40 Hz, and 140 Hz PRFs reliably increased the rates of calcium events associated with natural neuronal spiking. (a) Population average event density across all neurons before, during, and after US pulsed at various PRFs. Vertical colored lines represent US onset and offset. Neurons were sorted by event density difference during 1 s after versus before US onset. Neurons classified as activated by US are below the red dashed line. (Fisher’s test comparing the relative proportions of modulated neurons in stimulation vs sham conditions (see SI 2): 10 Hz US vs Sham *p* = 3.09 × 10^−15^, 40 Hz US vs Sham *p* = 1, 140 Hz US vs Sham *p* = 5.63 × 10^−26^, and 2 kHz US vs Sham *p* = 6.15 × 10^−09^). (b) Average modulated (orange, green, purple, and blue) and non-modulated (gray) population event density profiles. Shading represents mean event density ± SD. (c) Average peak-aligned calcium event waveform across events with peaks in the two seconds post-US (bottom, blue) compared to those during all other periods (top, gray). Shading represents mean ± SD. (d) Normalized Δ*F*/*F* heatmaps representing average calcium fluorescence activity during US. Vertical colored lines represent US onset and offset. Neurons were sorted by event density as in **2(a)**. Neurons classified as activated are below the red dashed line. (e) US-modulated and non-modulated population Δ*F*/*F* profiles. Colored lines represent modulated population profiles and gray lines represent non-modulated profiles. Shaded lines correspond to mean Δ*F*/*F* ± SD.

Of the 3,353 neurons imaged across all US parameters, 567 (16.9%) exhibited significantly increased calcium event density (figure 3(a)). US delivered at 10 Hz activated 162/861 neurons (18.82%) neurons, at 40 Hz activated 22/281 neurons (7.83%) neurons, and at 140 Hz activated 183/1019 neurons (17.96%) (figure 3(a), supplemental table 7). When pulsed at the higher PRF of 2 kHz, US activated 200/1192 neurons (16.78%). As expected, in modulated neurons, the average event density rose immediately following US onset, while the event density remained unchanged in non-modulated neurons (figure 3(b)). The average event fluorescence waveform during US closely matched the average event density waveform during non-US periods, indicating that US induces naturalistic calcium events related to neuronal spiking (figure 3(c)).

As a control, we recorded sham neural responses by omitting the US gel between the transducer and the mouse’s head, preventing acoustic wave propagation into the brain. This sham configuration allows us to rule out possible auditory activation due to pulsing US at the specific PRFs, given that mouse hearing is in the low ultrasonic range. Using the same statistical procedure comparing the sham-evoked response to the shuffled baseline distribution, we identified neurons activated during sham stimulation. Compiling all parameters, out of the 1915 sham neurons recorded, only 87 (4.54%) significantly increased calcium event density during sham US (SI. 2, supplemental table 8). The proportion of neurons modulated under the sham condition was significantly lower than the proportion modulated by US pulsed at 10 Hz, 140 Hz, and 2 kHz (figure 3(a), SI 2, supplemental tables 7 and 8, Fisher’s test, 10 Hz US vs Sham: *p* = 3.09 × 10^−15^, 140 Hz US vs Sham: *p* = 5.63 × 10^−26^, 2 kHz US vs Sham: *p* = 6.15 × 10^−09^). These results, along with our previous findings that a significantly smaller fraction of motor cortex neurons (0.25%) was activated by 2 kHz PRF US directed underneath the abdomen than to the head (42.9%), confirmed that the US-evoked motor cortex responses cannot be attributed to non-specific auditory or vestibular artifact [35]. However, 40 Hz US did not have a significant effect on modulation percentage compared to sham, likely due to smaller sample size (figure 3(a), SI 2, Fisher’s test, 40 Hz US vs Sham: *p* > 0.05). Together, these results demonstrate that transcranial US produced large amplitude and transient cellular calcium events consistent with those occurring during natural neuron spiking.

### 2.4. Transient activation of individual neurons during US does not produce long-lasting changes in network functional connectivity

**Figure 4.**
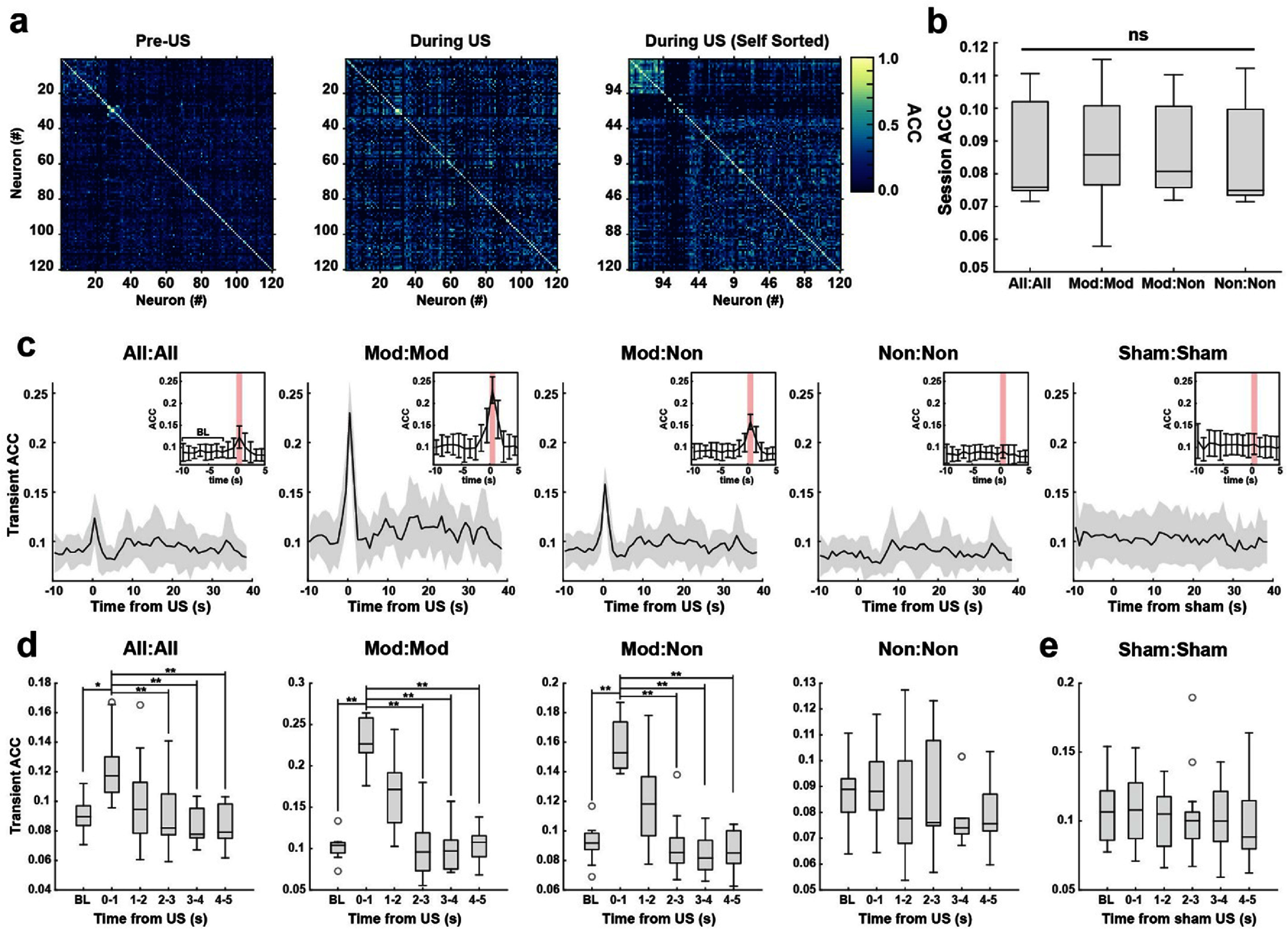
US-evoked network connectivity change is restricted to stimulation period. (a) Example session heatmap of ACC for cell pairs before US stimulation onset (pre-US) and during US at 10 Hz (during US). Left: ACC values were sorted with bispectral clustering to visualize subpopulations of more correlated cells. Middle: using the same sorting scheme as baseline, showing the loss of correlated clusters. Right: Resorted map during US stimulation period, showing a new cluster of correlated cells. (b) Session-wise averaged ACC throughout the full recording duration, for all cell pairs (‘All:All’), modulated cell pairs (‘Mod:Mod’), modulated and non-modulated cell pairs (‘Mod:Non’), and non-modulated cell pairs (‘Non:Non’). There was no significant difference for any cell pair group (Kruskal–Wallis, H(47) = 1.04, *p* = 0.79). (c) Time-course of ACC changes (‘Transient ACC’) over each second for each group of cell pairs. Traces are plotted as session-wise mean ± SD. Inset: bar plot with mean ACC ± SD used for statistical comparison. The first eight seconds of the trial-averaged sessions were averaged for statistical comparison and denoted as baseline ‘BL’. (d) and (e) Statistical comparison of transient ACC values during the first eight seconds (‘BL’) and post-US (d) or post sham-US (e). During US (0–1 s), transient ACC was significantly higher than BL for All:All, Mod:Mod, and Mod:Non groups (All:All Friedman test, H(59)= 25.31, *p* = 1.21 × 10^−4^, Nemenyi rank difference of US vs all other timepoints > CD for *α* = 0.05, Mod:Mod Friedman test, H(59) = 33.26, *p* = 3.35 × 10^−6^, Nemenyi rank difference of US vs all other timepoints > CD for *α* = 0.01, and Mod:Non Friedman test, H(59) = 28.91, *p* = 2.41 × 10^−5^, Nemenyi rank difference of US vs all other timepoints > CD for *α* = 0.01). There was no significant difference across timepoints for Non:Non or (e) sham cell pairs (Friedman test, *p* > 0.05).

The observed neural activity change in individual neurons, whether directly or indirectly responsive to US, is nonetheless associated with network changes, as activated neurons influence their downstream neuronal targets via synaptic connections. To estimate functional connectivity, we calculated the asymmetric correlation coefficient (ACC) using the calcium event traces of each neuron pair (see methods) (figures 4(a), (c) and (d)). We first examined ACC between pairs of US-activated neurons to understand whether functional connectivity contributes to their US-sensitivity. Interestingly, pairs of US-modulated neurons had similar ACC to all other cell pairs over the entire recording duration (figure 4(b), Kruskal–Wallis test, *p* = 0.79), suggesting that US-sensitive neurons are not particularly functionally connected under the awake head-fixed behavioral condition.

As US transiently increased calcium event rate in modulated neurons, we next evaluated the functional connectivity strength between pairs of modulated neurons (Mod:Mod), between pairs of modulated and non-modulated neurons (Mod:Non), and between pairs of non-modulated neurons (Non:Non). As expected, ACC between neuron pairs containing a modulated neuron (Mod:Mod and Mod:Non) transiently increased during US stimulation (0–1 s after onset), whereas ACC between two non-modulated neurons (Non:Non) did not change (figures 4(c) and (d), Friedman test, *p* < 0.001 for All:All, Mod:Mod, Mod:Non, and *p* > 0.05 for Non:Non and Sham:Sham). Across all cell pair subsets, increase in ACC dropped immediately at US offset and returned to baseline within a second of US offset (figure 4(d), post-hoc Nemenyi test, BL vs 1–2, 2–3, 3–4, and 4–5 s, non-significant for all cell pair subsets). Although we detected a significant ACC increase in all recorded neuron pairs during US stimulation, a similar increase was not present during sham stimulation (figure 4(e)). Further, US-modulated neurons demonstrated no spatial clustering (SI 3(a)–(c)). Thus, US induces a transient increase in network synchrony largely restricted to the stimulation period with minimal sustained effects.

### 2.5. Examining the differential effect of US pulsed at 10 Hz, 40 Hz and 140 Hz on parvalbumin-positive (PV) and parvalbumin-negative (non-PV) predominantly excitatory neurons

**Figure 5.**
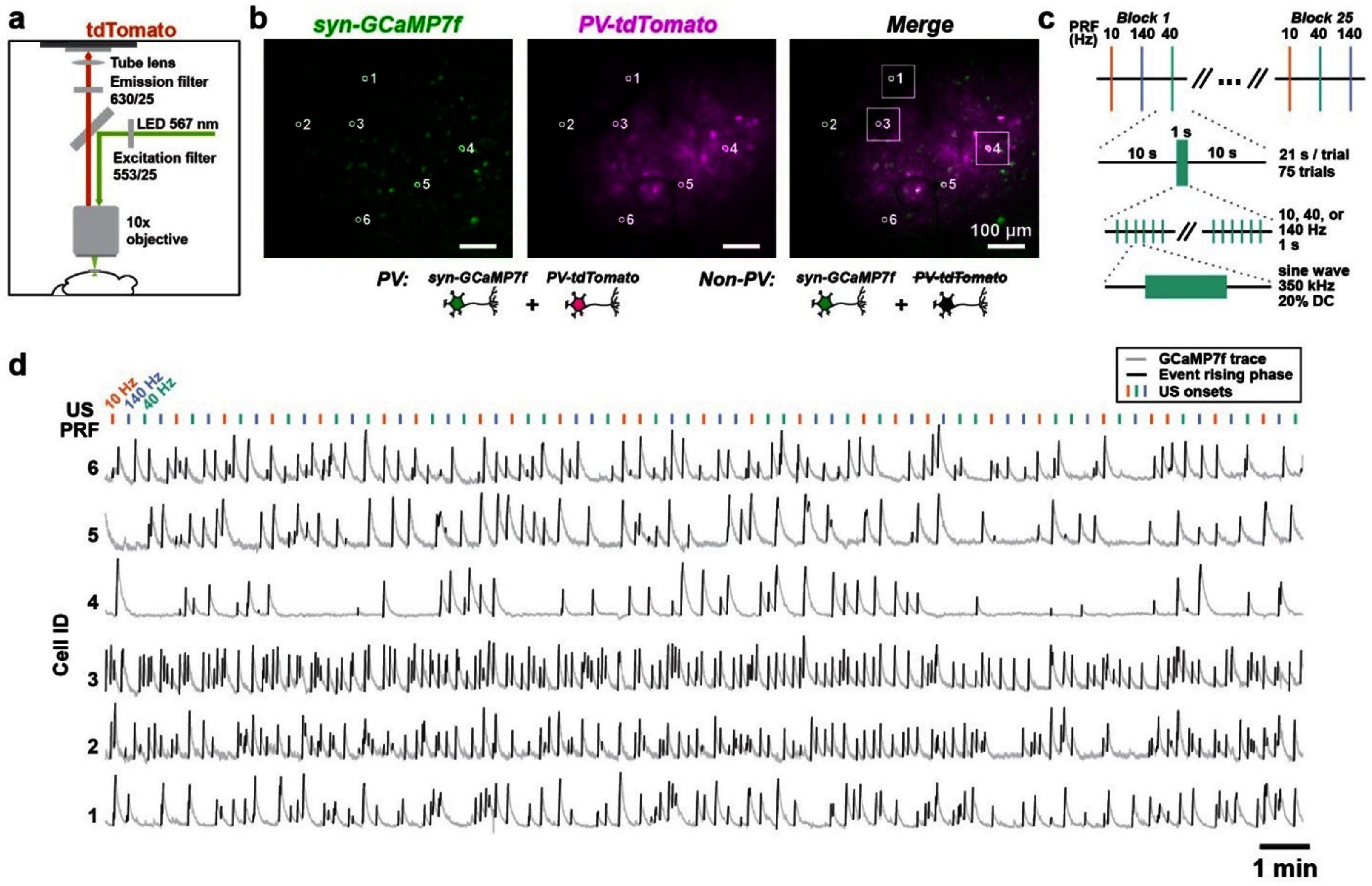
Direct comparison of the effect of different US PRF on the same neurons with or without PV expression. (a) Setup for imaging tdTomato (tdT) expressing PV interneurons. tdT expression was visualized using 567 nm LED excitation and 630/25 nm emissions. GCaMP7f was imaged with 460 nm LED excitation and 536/40 nm emission as illustrated in figure 1(a). (b) Left: An example GCaMP7f field of view, shown as fluorescence max-min projection image, with example individual neurons circled in white. Middle: Corresponding image of tdT for the same field of view. Right: Merged GCaMP7f and tdT images. PV neurons had colocalized tdT and GCaMP7f fluorescence. Neuron 4 was classified as PV, while example neurons 1, 2, 3, 5, and 6 were classified as non-PV. Boxed example neurons 1, 3, and 4 were modulated by US. All scale bars = 100 *µ*m. (c) US-Stimulation trial structure for alternating PRF experiments. Each recording contained 25 blocks, with randomly alternating 10 Hz, 40 Hz, and 140 Hz trials within each block. Each trial consisted of 10 s pre-US, 1 s US, and 10 s post US. 350 kHz US was pulsed at PRFs of either 10 Hz, 40 Hz, or 140 Hz at 20% duty cycle. (d) Calcium fluorescence traces for example neurons during the entire recording session. Gray lines represent GCaMP7f fluorescence and black lines represent calcium event rising phases. Colored lines above depict US delivered at randomly alternating PRFs. Scale bar is 1 min.

After observing reliable activation of neurons with transcranial US across PRFs, we sought to determine whether US pulsed at different PRFs produces distinct effects on the same neurons. As PV interneurons are known for their fast-spiking capabilities and contributes to 40 Hz gamma oscillations [40–44], we sought to compare PV interneuron and non-PV excitatory neuron responses across PRFs. We injected AAV9-syn-jGCaMP7f in the motor cortex of transgenic mice that selectively express the fluorophore tdTomato (tdT) in PV interneurons and then placed a glass imaging window above the pia for optical access of labelled neurons (figure 5(a)). This experimental preparation allowed us to simultaneously analyze US-evoked response in PV interneurons (expressing tdT) and non-PV neurons (tdT negative, figure 5(b)). Using this experimental configuration, we performed 21 recording sessions in 6 mice and imaged a total of 2,212 GCaMP7f positive neurons, including 269 (12.16%) PV cells, and 1,943 (87.84%) non-PV cells. The non-PV cell population is dominated by excitatory neurons as 80%–90% of neurons in the superficial motor cortex are excitatory pyramidal cells [59–61].

To understand how individual neurons respond to different PRFs, we compared the calcium responses evoked by US pulsed at 10 Hz, 40 Hz, and 140 Hz in the same neurons. As US-evoked GCaMP7f fluorescence returned to baseline levels within 10 s in our previous experiments (figures 2 and 3), we shortened the experimental trial structure to allow for 75 total stimulation trials within one 26 min recording period (figure 5(c)). With the increased statistical power, we divided the 75 trials into 25 blocks of alternating US PRFs at 10 Hz, 40 Hz, and 140 Hz. Each block consisted of three trials of randomly alternating PRFs, with 10 s pre-US, 1 s US stimulation, and 10 s recovery, for a total of 20 s between each US stimulation. We recorded GCaMP7f fluorescence during alternating PRF US and extracted fluorescence traces of individual neurons and identified calcium events (figure 5(d)).

### 2.6. Most neurons were activated by only one PRF regardless of PV expression

**Figure 6.**
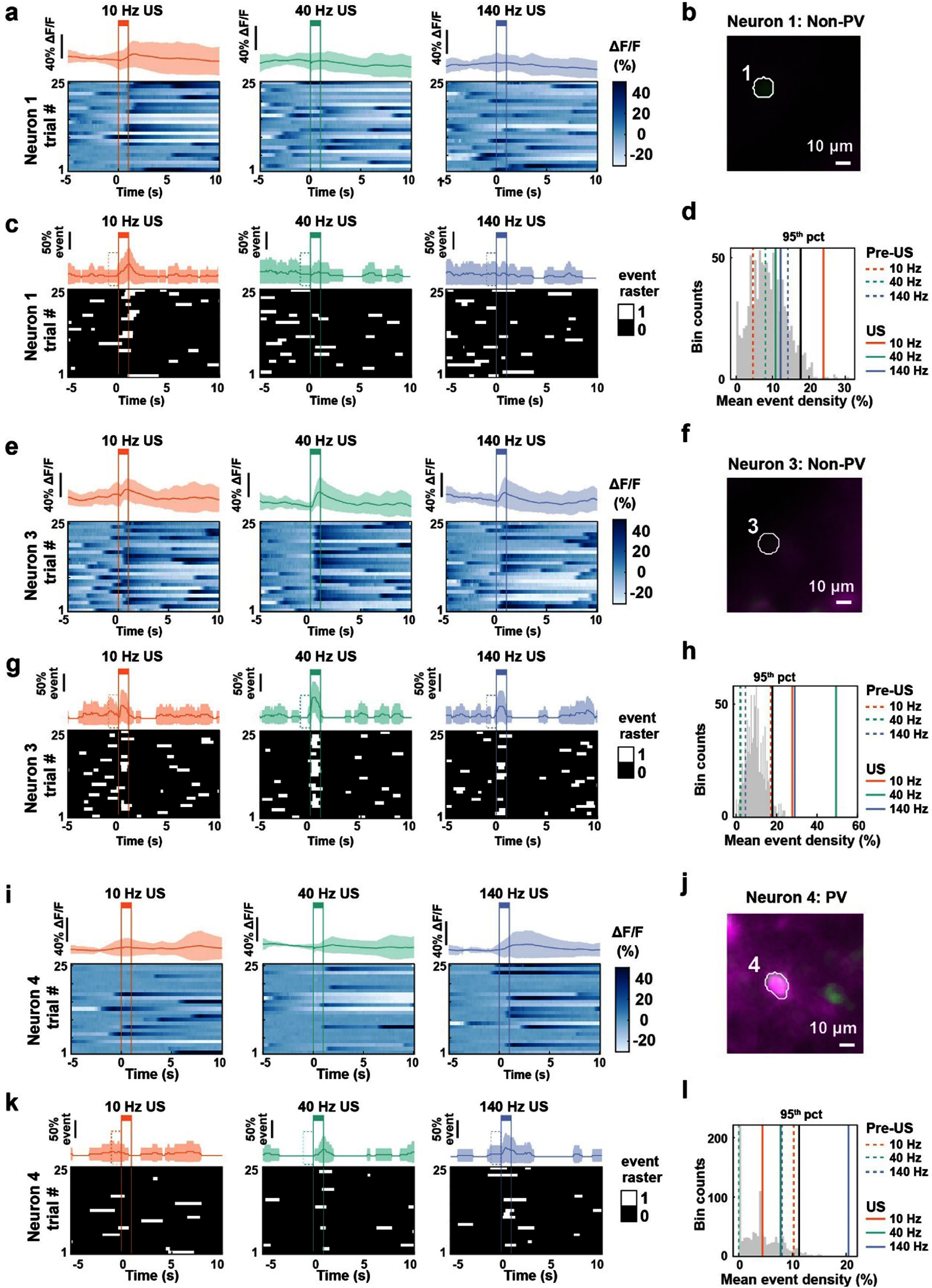
Individual neurons have different PRF preferences. (a), (e) and (i) Bottom: Normalized GCaMP7f Δ*F*/*F* heatmap aligned to US onset, plotted over 25 trials for example neurons #1 (a), #3 (e), and #4 (i) upon US at 10 Hz (left), 40 Hz (middle) and 140 Hz (right). Top: Trial-averaged GCaMP7f Δ*F*/*F* trace. Shaded region represents mean ± SD over 25 trials. Vertical colored lines correspond to US onset and offset respectively. (b),(f) and (j) Example maximum-minimum fluorescence images for the cells imaged. (b) non-PV neuron #1, (f) non-PV neuron #3, (j) PV neuron #4. Scale bar = 10 *µ*m. (c), (g) and (k) Bottom: Binarized event traces over 25 trials, with white corresponding to time points of event rising phases and black everywhere else. Vertical lines represent US stimulation onsets and offsets. Top: Trial-averaged event density trace. Shaded region represents mean ± SD. Dashed box prior to US depicts one second pre-US. (d),(h) and (l) Illustration of statistical shuffling test used to determine neuron #1 (d) being modulated only by 10 Hz US, neuron #3 (h) being modulated by 10 Hz, 40 Hz, and 140 Hz US, and neuron #4 (I) being modulated only by 140 Hz US. Shuffled baseline event density is shown as a gray histogram with the 95-percentile indicated by solid black line. The observed event densities during US are shown as solid orange (10 Hz), green (40 Hz), or purple (140 Hz) lines, and the observed event densities pre-US are shown as dashed orange, green, or purple lines.

Testing multiple PRFs on the same neuron allowed us to directly compare the sensitivity of each neuron to different US PRFs (figures 6(a)–(l)). To determine whether a neuron was activated by a specific US PRF, we compared the mean calcium event density during US of a given PRF (figures 6(c), (g) and (k)) to the shuffled baseline event rate distribution (figures 6(d), (h) and (l)). To generate the shuffled baseline distribution for alternating PRF experiments, we calculated the mean event rate from 25 randomly selected 1 s periods during the full recording excluding 5 s after all US onsets and repeated this procedure 1000 times. Neurons were deemed responsive to a specific PRF if the US-evoked event density was greater than the 95th percentile of the shuffled baseline distribution. To avoid false positive identification, neurons with significantly elevated event density during the 1 s window before a given US period were excluded from being considered modulated by that PRF.

**Figure 7.**
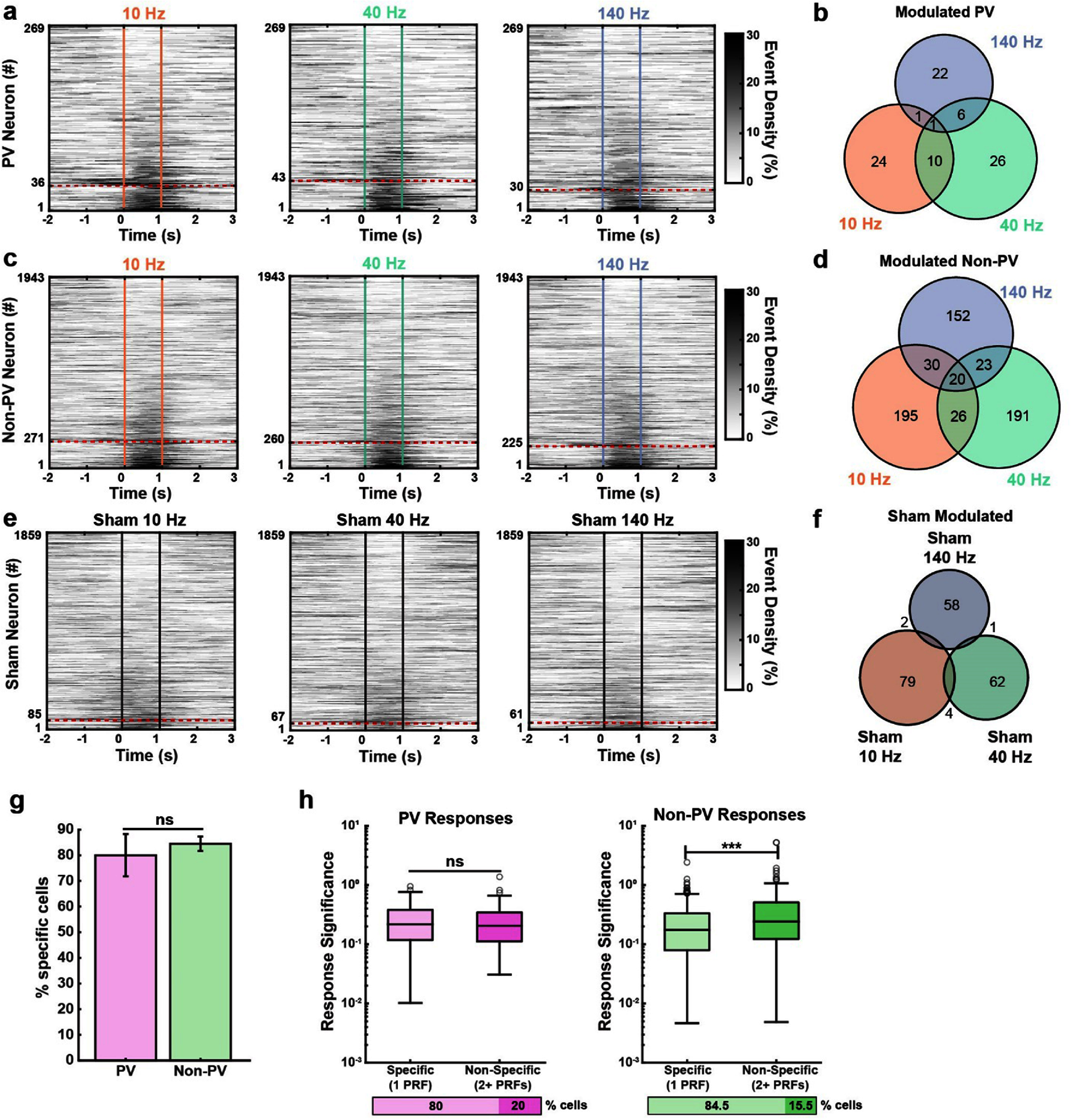
A similar proportion of largely non-overlapping cells were activated by US in both PV and non-PV populations. (a), (c) and (e) Population average event density across (a) PV neurons, (c) non-PV neurons, and (e) sham condition. The colored lines represent US onset and offset. Neurons were sorted by event density difference during 1 s after versus before US onset. Neurons classified as activated by US are below the red dashed line. The proportion of modulated neurons in each sham condition was significantly lower than the corresponding stimulation condition (Fisher’s Test, 10 Hz US vs Sham: *p* = 5.27 × 10^−25^, 40 Hz US vs Sham: *p* = 3.03 × 10^−21^, and 140 Hz US vs Sham: *p* = 2.70 × 10^−24^). *N* = 21 imaging sessions for stimulation and *N* = 13 imaging sessions for sham. (b), (d) and (f) Venn diagram of the cell population modulated by the specific RPFs. The number in each region corresponds to the number of cells modulated for that specific group. (g) Proportion of cells with responses to one PRF. Error bars represent population 95% confidence intervals. There was no significant difference between the proportion of PRF-specific cells between PV and non-PV populations (Fisher’s test, *p* = 0.28). (h) Response significance of cells responding to one PRF (‘specific’) versus to two or more PRFs (‘non-specific’). There was no significant difference in the response significance between non-specific and specific PV cells (Wilcoxon rank-sum test, *p* = 0.75, *Z* = 0.31). The non-specific non-PV cells had a significantly higher response significance than the specific non-PV cells (Wilcoxon rank-sum test, *p* = 2.27 × 10^−6^, *Z* = −4.73). *Y*-axis scale is logarithmic.

**Figure 8.**
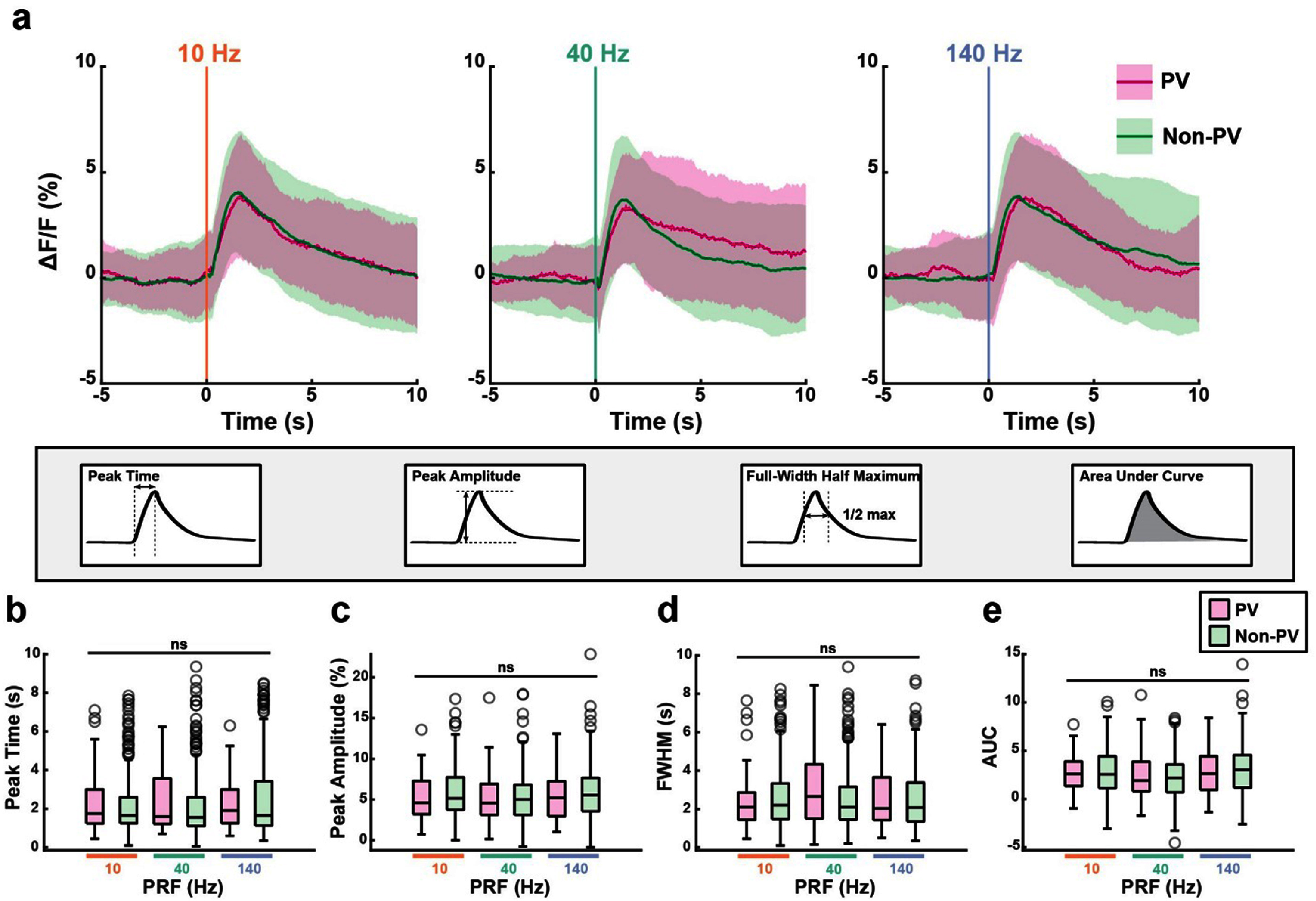
The temporal dynamics of US-evoked responses are similar across cell types and PRFs. (a) Average calcium fluorescence traces for 10 Hz, 40 Hz, and 140 Hz modulated PV and non-PV neurons. Shading represents mean ± SD. (b)–(e) Modulated cell’s Δ*F*/*F* calcium response profiles, characterized as time to peak (b), response amplitude (c), full width at half maximum (d) area under curve in the 5 s post-US (e). There is no difference across comparisons, except for area under curve (GLM, Deviance test, time to peak: *p* = 0.18; response amplitude: *p* = 0.23, full width at half maximum: *p* = 0.72; area under curve: *p* = 0.045).

We found that both cell populations contained a sizable fraction of neurons that increased event density during US stimulation (figures 7(a)–(d)). Of the 269 PV neurons recorded, a similar proportion (11.2%–16.0%) were activated by US pulsed across PRFs (figure 7(a), SI 4(a), supplemental table 9, Chi-Square test, *p* = 0.26). However, the activated neurons by different PRFs were largely non-overlapping (figure 7(b)). Similarly, the fraction of US-activated non-PV excitatory neurons was also similar across the three PRFs tested (11.6%–14%) (figure 7(c), SI 4(b), supplemental table 10, Chi-Square test, *p* = 0.07) and largely non-overlapping (figure 7(b)). There was no difference between the fraction of activated PV and non-PV neurons for any of the PRFs (figures 7(a) and (c)), Fisher’s test, 10 Hz PV vs Excitatory *p* = 0.85, 40 Hz PV vs Excitatory *p* = 0.26, and 140 Hz PV vs Excitatory *p* = 0.92). Further, US-evoked calcium fluorescence increase was also similar between these two cell populations, both exhibiting a sharp rise followed by a gradual decay, characteristic of GCaMP7f spike-related calcium event profiles (figure 8(a), SI 4(a) and (b)). To estimate the response latency, we averaged the calcium responses of modulated neurons across all PRFs and found a population response latency of 200 ms, corresponding to the time when the average fluorescence was greater than two standard deviations above the 10 s baseline.

As a control, we employed sham stimulation by removing the US gel between the transducer and the mouse head as described previously (figures 7(e) and (f)). We recorded a total of 1859 neurons during sham stimulation across 13 sessions in 6 mice. Sham stimulation activated 3.3%–4.6% of neurons at the PRFs tested (figure 7(e), supplemental table 11), significantly less than that activated by US (figures 7(a), (c) and (b)), Fisher’s test, US vs sham, *p* < 0.001 for 10 Hz, 40 Hz, and 140 Hz).

Interestingly, we noticed that while most neurons were responsive to one PRF (‘specific’ responders, examples shown in figures 6(a)–(e) and (k)–(o)), a small fraction responded to multiple PRFs (‘non-specific’ responders, examples in figures 6(f)–(j)). Of the 269 PV cells recorded, 72 (26.76%) were specific responders, and 18 (6.7%) were non-specific responders (figure 7(b)). Of the 1943 non-PV excitatory cells recorded, 538 (27.69%) were specific responders and 99 (5.1%) were non-specific responders (figure 7(d)). There was no difference between the fraction of specific and non-specific responders between the two cell groups (figure 7(g), Fisher’s Test, PV vs non-PV, *p* = 0.28). Together, these results demonstrate that most modulated neurons were selectively activated by one PRF, but not the other two. This result further ruled out the possibility of auditory nerve activation effect induced by US via non-specific cochlear fluid vibration as suggested by Guo *et al*, where US delivered at different PRFs from 10 Hz to 1.5 kHz drove auditory nerve activation similarly [45].

While most activated neurons were specific responders, we also observed some non-specific responders. It is possible that non-specific responders have higher sensitivity to US due to their biophysical properties or mechanosensitive channel expression profiles. If these non-specific responding neurons were more sensitive to US, one would expect greater evoked responses to a given PRF, in addition to being responsive to multiple PRFs. Thus, we calculated US-evoked response significance, defined as the difference between the observed rate minus the 95th percentile of the corresponding baseline shuffled rate distribution divided by the 95th percentile of the shuffled rate distribution. For the PV population, we found no difference in response significance between specific versus non-specific responders (figure 7(h) (left), Wilcoxon rank-sum test, *p* = 0.75). In contrast, for the non-PV population, non-specific responders had significantly higher response significance than specific responders (figure 7(h) (right), Wilcoxon rank-sum test, *p* = 2.27 × 10^−06^), suggesting a higher sensitivity of non-specific responders to US. This is consistent with the observation that excitatory neurons generally have greater mechanosensitive ion channel expression than GABAergic neurons (figures 1(b), (f) and (g), SI figure 1). As the non-PV population includes both excitatory cells and inhibitory interneurons of various subtypes (figure 1(b)), it is also possible that the difference in US-evoked response significance reflects the heterogeneity of the non-PV population.

### 2.7. US-evoked calcium response profiles are similar in PV and non-PV populations across all PRFs tested

A few previous studies described small but nonetheless significantly different population responses to US between PV cells and non-PV cells [7, 16]. At the individual neuron level, we detected a similar proportion of activated cells between PV and non-PV populations. Thus, we further compared the population response profiles between PV and non-PV neurons (figure 8(a)). We found no difference in US-evoked population calcium response peak timing, peak amplitude, and full-width half maximum between the two cell populations (figures 8(b)–(d), generalized linear model (GLM), deviance test *p* > 0.05). Although the area under curve (AUC) model was slightly different than the GLM intercept only model (deviance test, *p* = 0.0488), none of the model coefficients, including the interaction term, were significant, indicating that the model only weakly predicts the experimental values (figure 8(e), GLM coefficients *p* > 0.05).

There were also no major differences between the transient change in correlation coefficients among PV cell pairs, non-PV cell pairs, and PV:non-PV cell pairs (SI 5(a)–(f)). As with the population of synapsin-GCaMP7f recorded previously (figure 4(e)), we found US transiently increased the correlations between cell pairs containing modulated neurons (SI 5(b), (d) and (f)). Interestingly, after the transient increase in correlation coefficients, there was a small but significant decrease in correlation between non-modulated non-PV cell pairs and PV:non-PV cell pairs, but not in PV:PV pairs (SI 5(b), (d) and (f)). This delayed change in network correlation supports a network effect evoked by US. Thus, US-evoked calcium responses exhibited similar profiles between activated PV and non-PV populations. Taken together, cortical PV neurons and non-PV predominantly excitatory cells responded similarly to US stimulation at 10 Hz, 40 Hz, and 140 Hz, with a subset of each cell type preferentially responding to a single PRF. These results provide direct experimental evidence that heterogeneity among individual cells, rather than systemic variation between cell types, dominates the effect of US neuromodulation.

## 3. Discussion

US stimulation serves as an external input to a neuron. Thus, the effect of US neuromodulation critically depends on the intrinsic cellular properties of neurons. Since the speed of neuronal activity is limited to a couple hundred hertz and many neurons are more sensitive to certain frequency ranges, we examined whether delivering US at physiologic PRFs would selectively engage distinct neurons in the awake mammalian brain. We analyzed US-evoked cellular responses in thousands of individual cortical neurons using GCaMP7f calcium imaging in awake mice. We delivered 0.35 MHz US transcranially at the physiologically relevant frequencies of 10 Hz, 40 Hz, and 140 Hz and the conventional supraphysiologic PRF of 2 kHz. We found that all PRFs reliably evoked intracellular calcium events with similar characteristics to those naturally occurring during neuronal spiking. The evoked cellular changes are accompanied by a transient increase in network synchrony that diminished shortly after the stimulation. At the population level, evoked responses were similar across PRFs and between PV positive fast spiking interneurons and non-PV putative excitatory neurons. Most intriguingly, comparing the effect of 10 Hz, 40 Hz, and 140 Hz US on the same neurons, we found that most neurons were preferentially activated by a single PRF, highlighting that different subsets of neurons could be recruited by combining PRFs. As most US-activated neurons were only responsive to a single PRF and sham stimulation failed to activate neurons, the observed effects cannot be explained by nonspecific auditory or vestibular effects.

While the underlying mechanisms behind US’s engagement of cellular signaling are unclear, mechanosensitive channels are likely vital in translating US-mediated cellular effects to downstream signaling pathways [14, 16, 19, 24, 62]. We thus analyzed the expression patterns of major mechanosensitive channels using the Allen Brain Institute’s single cell RNA sequencing data obtained from human and mice brain tissue. We found that even though several mechanosensitive channels exhibit higher expression in excitatory neurons than inhibitory neurons, expression variation between individual neurons appears much more prominent than between cell types. This is consistent with our cellular calcium imaging observation that US-mediated effects are more heterogenous between individual cells than canonical cell types.

Most *in vivo* US neuromodulation studies have used methods to observe bulk neuronal responses such as LFP, EEG, and EMG [63, 64]. In contrast, we here assessed the cellular effect across individual neurons and discovered that many neurons were preferentially activated by one PRF, but not the other two. Although motor cortex neurons may be activated by a startle-reflex at stimulation onset [45, 65], the PRF discrimination of individual neurons cannot be explained by the broadband frequency components of the square-wave pulse onset underlying all PRFs. Similarly, the fact that many neurons responded to only one of the three PRFs is not consistent with US-induced non-specific cochlear fluid vibration suggested in previous studies [45, 65], as different PRFs are expected to vibrate the cochlear fluid similarly. Further, as mice hear similar US pulse patterns in our sham condition without US gel coupling, the lack of neuronal activation in the sham conditions further ruled out the possibility of indirect auditory activation. Finally, we detected an activation latency of 200 ms, which again is inconsistent with the long-latency effect of indirect auditory activation of motor cortex neurons described previously [65]. Instead, our results are consistent with the direct neuronal effects of US on individual neurons, as also reported in the hippocampal CA1 in awake mice with sub-50 ms activation latency [35], in deafened mice [66], in neuron cultures [19, 67], and in simple nervous systems [31]. Thus, the observed PRF-dependent US activation of individual neurons cannot be due to indirect auditory or sensory pathway activation.

US activation of individual neurons involves direct engagement of mechanosensitive cellular elements, which subsequently induces intracellular cascades, recruiting voltage gated sodium and potassium channels and altering neural activity [14, 16, 19, 24, 62]. The observed intracellular calcium increases upon US stimulation are thus expected to involve cellular signaling dependent on both plasma membrane biophysical properties (i.e. mechanosensitive ion channels) and cellular microenvironment (e.g. cell position within US wave, local chemical gradients). Our in-depth analysis of the Allen Brain Institute’s datasets revealed that many mechanosensitive channels are broadly expressed in the brains in mice and humans. Individual channels’ expression levels vary widely between brain regions. Further, the variation of channel expression is more striking among individual cells, even within a given transcriptomically defined cell type. The heterogeneity of mechanosensitive protein expression could thus result in varying US sensitivity across brain regions, cell types, and more prominently among individual cells. Consistent with this idea, we found that US sensitivity was highly heterogeneous across individual neurons, and there were no major differences between PV-positive interneurons and PV-negative predominantly excitatory neurons, suggesting that US effect is dominated by cellular signaling variations among individual cells. Adding to the variable response of neurons, US activation of mechanosensitive TRPA1 channels in astrocytes has shown to activate nearby neurons through astrocytic glutamate release [68].

Both *in vivo* and *in vitro* US neuromodulation studies implementing a wide array of parameters have demonstrated that US mediated neural responses depend on a variety of factors including brain regions [25, 35] and cell types [34, 37]. Interestingly, US delivered at 10 Hz, 40 Hz, and 140 Hz activated comparable proportions of neurons with similar response profiles, even though many neurons were preferentially activated by only one PRF. These results support the idea that physiologically relevant PRFs differentially impact the coupling of mechano-sensing elements and downstream cellular signaling to mediate US neuromodulation. Interestingly, we did not notice any difference in US-mediated effect between PV and non-PV populations at 10 Hz, 40 Hz, or 140 Hz PRFs. Even though PV interneurons are broadly linked to 40 Hz gamma rhythms, we detected no difference in their evoked calcium responses compared to non-PV cells across PRFs, including 40 Hz. Some recent studies reported slight differences in US-evoked responses between fast-spiking PV cells and excitatory neurons [34, 37]. Our current study’s estimated acoustic pressure amplitude *in situ* was ∼0.29 MPa, lower than the 0.6 MPa used in Murphy *et al* [34], potentially contributing to the observed similar effects between cell types. We also found that US-evoked calcium events exhibited similar characteristics as those naturally occurring during increased spiking in the absence of US stimulation. However, as calcium imaging is slow, we may have missed the subtle differences in US-evoked spiking activity captured by extracellular recordings of putative PV positive fast spiking cells and putative excitatory broad spiking cells [37].

Previous *in vivo* studies detected diverse neural responses to US across multiple brain regions [25, 35, 69]. However, none directly compared the neuronal response to multiple parameters at the single cell level across different cell types. We found that most neurons were preferentially activated by a single PRF. Combining PRFs, such as the three tested here, could thus enhance the efficacy of US neuromodulation by recruiting different subsets of neurons in clinical applications. With future investigation on the effect of adjusting or combining PRFs to target desired neuronal subgroups, it may be possible to design personalized medicine opportunities. While the majority of PV and non-PV neurons selectively responded to a single PRF, some responded non-selectively to two or more. Heterogenous expression of mechanosensitive channels and membrane ion channels with varying resonant frequencies may contribute to PRF dependent activation between different neurons within the same cell type. Despite consistent calcium dynamics observed in both responding cell types, only the non-PV population, but not the PV population, exhibited variations in response significance when comparing selective and non-selective responders. As the non-PV cell population comprises mainly pyramidal neurons, heightened response significance in non-PV cells is consistent with higher mechanosensitive channel expression observed in our single cell gene expression analysis.

Numerous factors influence neuronal excitability including instantaneous membrane potential, network activity, and channel distribution. Using statistical resampling tests, we identified about 10%–20% of neurons were activated by a single PRF. However, US evoked responses within the same neurons exhibited large variations across trials, confirming US’s weak modulatory effect. One potential explanation for this weak effect is that US depolarizes neurons through mechanosensitive channel activation, thus indirectly increasing spiking probability via downstream signal amplification by voltage and calcium gated channels [19, 62]. Nonetheless, the overall weak effect is consistent with the behavioral state, brain region and pulse pattern dependent effect of US observed across various studies [34, 35, 37, 69]. Future studies looking into mechanosensitive channel sensitivity to US and its role in engaging cellular signaling pathways that govern neuronal excitability will further clarify the nature of US stimulation and frequency response bias. Finally, while this study focuses on US PRF’s impact on neuronal responses, the effects of additional parameters, including fundamental frequency, acoustic intensity, and stimulation duration, should also be investigated in single cells. For example, increasing US intensity and stimulation duration has been shown to improve US efficacy in evoking motor responses, while increasing fundamental frequency reduces these effects [70]. Systematically exploring the impact of these parameters on individual neuronal responses will be a necessary step in optimizing stimulation protocols and cultivating a mechanistic framework for US neuromodulation.

## 4. Methods

### 4.1. Allen brain institute gene expression analysis

Data are from Allen Institute for Brain Science, https://brain-map.org/atlases-and-data/rnaseq/human-m1-10x and https://brain-map.org/atlases-and-data/rnaseq/mouse-whole-cortex-and-hippocampus-10x. Uniform Manifold Approximation and Projection representation of the mouse single cell sequencing results were created with Cytosplore Viewer, https://viewer.cytosplore.org/. To normalize expression levels for individual cell comparisons, raw unique molecular identifier (UMI) counts were divided by the total UMI count for the given cell, multiplying by a scaling factor of 100 000, adding 1, and performing a log-2 transform. Expression level distribution overlap is measured with Cliff’s delta ($\delta $), with $\left| \delta \right| &lt; 0.147$ considered negligible, $0.147 \unicode{x2A7D} \left| \delta \right| &lt; 0.330$ considered small, $0.330 \unicode{x2A7D} \left| \delta \right| &lt; 0.474$ considered medium, and $\left| \delta \right| \unicode{x2A7E} 0.474$ considered large.

### 4.2. Animal preparation

All animal experimental procedures were approved by the Boston University Institutional Animal Care and Use and Biosafety Committees. When possible, mice were group housed prior to surgery and single-housed post-surgery. Enrichment was provided with Igloos or running wheels. Animal facilities were maintained around 70°F and 50% humidity and were kept on a 12 h light/dark cycle. We used a total of 16 adult mice, including 10 C57BL6 mice (9 female and 1 male) and 6 PV-tdT transgenic mice that were generated by crossing B6.Cg-Gt(ROSA)26Sor^tm14(CAG-tdTomato)Hze^/J (Jax, stock number: 007914) with B6.129P2-Pvalb^tm1(cre)Arbr^/J (Jax, stock number: 017320) mice (3 male and 3 female) (Jackson Laboratory, Bar Harbor, ME). Mice were 8–16 weeks old at the time of surgery.

Animal preparations are identical to those described previously [35, 71]. Briefly, under isoflurane general anesthesia, we first performed a craniotomy ∼3 mm in diameter over the right motor cortex (AP: 1.75 mm, ML: 1.75 mm). We then infused a total of 0.5 ul of AAV9-syn-GCaMP7f (titer 2.3 × 10^13^ GC ml^−1^, Addgene 104488-AAV9, Watertown, MA) at two separate locations within the craniotomy, about 180-nm below the dura, at 80 nl/min using a World Precision Instruments NanoFil syringe (NANOFIL) with a 36-gauge bunt needle (NF36BL-2). Following injection, the injection needle was left in place for 10 min at each site to allow AAV diffusion. We then placed a cover glass (no. 0, OD: 3 mm, Deckgläser Cover Glasses, Warner Instruments, 64-0726 (CS-3R-0)) over the viral injection site and fixed it in place using the UV curable glue (Tetric EvoFlow (Safco, Buffalo Grove, IL). Metabond Quick Adhesive Cement System (SKU: S380 Parkell, Edgewood, NY) was then used to cover any exposed skull, followed with dental cement (5145, Stoelting, Wood Dale, IL) to fix a custom metal head-bar posterior to the motor cortex near lambda. Mice were administered preoperatively with buprenex (0.1 mg kg^−1^) or with sustained release buprenorphine (3.25 mg kg^−1^, Ethiqa XR, Fidelis Pharmaceuticals, North Brunswick Township, NJ) postoperatively to provide a minimum of 48 h analgesia. Following surgery, mice were given a 2–3 week recovery period before recording sessions began.

### 4.3. Calcium imaging and US stimulation

Mice were habituated for one week and then imaged using a custom wide-field microscope equipped with a 10x objective at NA 0.28 (10x M Plan APO-378-803-3, Mitutoyo, Takatsu-ku, Kawasaki, Kanagawa, Japan). A 460 nm LED (LZ1-00B200; LedEngin, San Jose, CA) with an excitation filter at 470/50 nm (FF01-468/553-25; Semrock, Rochester, NY) was used to excite GCaMP7f. GCaMP7f fluorescence emissions then passed through a dichroic mirror (FF493/574-Di01-25x36; Semrock, Rochester, NY) and were filtered at 536/40 nm (FF01-512/630-25; Semrock, Rochester, NY). GCaMP7f imaging was performed at 20 Hz with a sCMOS camera (Hamamatsu ORCA Fusion C14440-20UP; Hamamatsu Photonics K.K., Shizuoka, Japan).

Mice were positioned below the objective and head-fixed on a 3D printed platform, directly above a planar US transducer with 350 kHz center frequency (GS350-D13, Ultran, State College, PA). The chin of the animals was placed at the center of the US transducer, and the space between the chin and the transducer was filled with US gel (Aquasonic Clear^®^ US Gel 03-08_BX, Parker Laboratories, Fairfield, NJ) to ensure US propagation to the brain. Sham stimulations were performed without US gel between the chin and the transducer.

Two US stimulation protocols were used in this study. For the first protocol, we recorded long duration trials while delivering US at a single PRF. For these experiments, each recording session had 20 imaging trials, with each trial lasting for 50 s containing a 10 s period before US followed by 1 s of US stimulation and 39 s recovery, for a total inter-stimulation interval of 49 s. US PRF of 10 Hz, 40 Hz, or 140 Hz at 20% DC or 2 kHz at 42% DC was used. There was a 0.5 s pause between each trial, and GCaMP7f fluorescence traces were interpolated to account for missing data points between trials. For the second protocol, we recorded shorter duration trials with PRF alternating between 10 Hz, 40 Hz, and 140 Hz at 20% DC during each session. Specifically, each recording session consisted of 25 blocks, with each block containing a randomly permuted PRF combination of 3 trials at 10 Hz, 40 Hz, and 140 Hz respectively. Thus, each recording contained 75 trials, with 25 trials per PRF. Each trial began with a 10 s period without US followed by 1 s of US stimulation and 10 s post-stimulation. There was a 0.5 s pause between each trial to maintain signal alignment, and the resulting GCaMP7f fluorescence traces were interpolated to account for missing data points.

US parameters including PRF, stimulation duration, and US onset time were programmed in MATLAB (MATLAB r2021b, MathWorks, Natick, MA), which sent TTL pulses through a NI-DAQ multifunction I/O system (PCIe-6321, National Instruments, Austin, TX) to trigger camera acquisition and US pulses. For US stimulation, MATLAB programmed TTL pulses triggered a function generator (33220A, Keysight, Santa Rosa, CA) to generate a 350 kHz sine wave at the desired repetition frequency and duty cycle. The function generator output was connected to an amplifier (AG1006, T&C Power Conversion, Rochester, NY) to amplify the voltage signal at 350 kHz. The amplified voltage signal was sent to the US transducer, which converted the voltage to an acoustic wave. OpenEphys (Open Ephys Acquisition Board v2.4, OpenEphys, Atlanta, GA) acquired individual imaging frame timestamps from the camera and all TTL triggers from the NI-DAQ to allow for offline alignment between US stimulation onsets and calcium imaging frames.

### 4.4. Hydrophone calibration and intensity calculations

Acoustic intensity measurements were performed with a needle hydrophone calibrated between 0.25–1 MHz (HNR-1000, Onda Corporation, Sunnyvale, CA) and connected to a pre-amplifier (AH-2010, Onda Corporation). The hydrophone was positioned above the 350 kHz US transducer in degassed water. The free-field spatial peak of the US beam was found by spatially translating the hydrophone in the *x, y*, and *z* planes while monitoring peak-peak voltage on an oscilloscope. After finding the spatial peak, the hydrophone waveform was recorded with a high-speed 14-bit digitizer card (Octave CSE8325, GaGe by VITREK, Lockport, IL). Voltage waveforms were converted to pressure with the calibrated sensitivity at 350 kHz adjusted for the preamplifier (7.234 × 10^−6^ V Pa^−1^). We estimated free-field spatial-peak pulse-average intensity (*I*_SPPA_) with the following equation: ${I_{{\text{SPPA}}}} = \frac{{p_{{\text{sp}}}^2}}{{2Z}}$, where ${p_{{\text{sp}}}}$ is the spatial peak pressure amplitude and the acoustic impedance of water is $Z \approx 1.5 \times {10^6}$ Rayls. Spatial-peak, temporal average intensity was then calculated by multiplying ${I_{{\text{SPPA}}}}$ by the duty cycle of stimulation.

### 4.5. US acoustic pressure simulation

Acoustic pressure was simulated with the k-Wave acoustic modeling toolbox in MATLAB as described previously in Tseng *et al* [35]. Briefly, acoustic pressure was simulated within a three-dimensional representation of a C57BL/6 mouse skull (adapted from Chan *et al*) [57]. The skull model was modified to include a craniotomy and a glass coverslip above the motor cortex, and two air pockets were added in the mouth to simulate acoustic propagation from below the chin. Both soft tissue and bone were considered homogeneous with bulk acoustic properties, and the mouse head model was surrounded by air at 50% humidity and 25 °C. As in the experiments, the mouse head was coupled to the acoustic source via US gel, which was modeled as water. The simulation’s acoustic source was a 12.36 mm diameter single element planar US transducer. The k-Wave computational grid was discretized at 0.12 mm and the transmitted US was a 25-cycle burst at 350 kHz with peak pressure of 522 kPa, as estimated with hydrophone measurements. The k-Wave simulation estimated the maximum instantaneous acoustic pressure and intensity throughout the brain during US stimulation. To validate simulation results, we measured the acoustic pressure in a mouse skull filled with US gel with a hydrophone.

**Table:** 

Medium	${\boldsymbol{c}}\left( {\mathbf{m}{\mathbf{s}^{ - 1}}} \right)$	${\boldsymbol{\rho}} \left( {\mathbf{kg}\;{\mathbf{m}^{ - 3}}} \right)$	${\boldsymbol{\alpha} _0}\left( {\mathbf{dB}}/{\mathbf{MH}}{\mathbf{z}^\gamma}/{\mathbf{cm}} \right)$	${\boldsymbol{\gamma}} $
Water	1482	1000	0.0022 [72]	2 [72]
Brain & soft tissue	1562 [73]	1035	0.58 [73]	1.3 [73]
Bone	2850 [74]	1732 [74]	3.54 [73]	0.9 [73]
Air	343	1.20 (x100)^a^	12 [72]	2 [72]
Glass	4540	2000	0.001 737 [75]	2 [75]

^a^ As recommended by k-Wave, the modeled density of air was raised 100x to prevent simulation convergence errors due to high acoustic impedance mismatch. Despite these changes, the reflection coefficient remained approximately −1, as expected.

### 4.6. Calcium imaging ROI segmentation and GCaMP7f trace extraction

Calcium videos were first motion corrected as described in Tseng *et al* [35], and then regions of interest (ROIs) corresponding to individual neurons were automatically segmented using a custom deep learning network followed by manual inspection to remove ROIs that do not exhibit neuronal morphologies. Additional ROIs missed by the algorithm were added during manual inspection in MATLAB. The GCaMP7f trace for each neuron was calculated as the mean intensity of all pixels within a ROI minus the mean pixel intensity of the surrounding ‘donut’ masks (inner radius 15 pixels from ROI centroid and outer radius 50 pixels from ROI centroid) to remove potential local background activity. Pixels within ROIs were excluded from the background donut mask. GCaMP7f traces were interpolated to 20 Hz using shape-preserving piecewise cubic interpolation and linearly detrended to remove photobleaching effects. Finally, GCaMP7f traces were normalized between 0 and 1 by subtracting the minimum trace value and dividing by the fluorescence intensity range. To ensure similar values in the 10 s pre-US period throughout trials, the final Δ*F*/*F* value was calculated by subtracting the average value of the 10 s pre-US period for each trial from that trial’s trace.

### 4.7. Parvalbumin (PV) positive interneuron detection

To identify PV cells among the recorded GCaMP7f expressing neurons, we captured tdT fluorescence at 100 ms exposure, immediately prior to GCaMP7f recording. From the tdT fluorescence image, we marked matching vascular landmarks in the tdT images and GCaMP7f images and warped the tdT image to match the GCaMP7f vascular landmarks (MATLAB estimateGeometricTransform and imwarp functions). Next, we identified tdT positive cells (tdT ROIs) using the neuron segmentation method detailed above to identify GCaMP7f positive neurons (GCaMP7f ROIs). We then identified PV cells as GCaMP7f ROIs with >50% overlap with tdT ROIs.

### 4.8. Calcium event detection

GCaMP7f calcium events were detected using the frequency spectral profile of their sharp rise in fluorescence, as described previously [71]. Briefly, each GCaMP7f trace was smoothed with a 1 s sliding window (MATLAB movmean). Next, the continuous multi-taper time-frequency spectrum was found for each smoothed GCaMP7f trace (Chronux Toolbox in MATLAB, http://chronux.org). Potential event peaks were found as points in the frequency spectrum above the median normalized power. Potential events with rise times greater than 100 ms and amplitudes greater than 2.5x the pre-event standard deviation were classified as calcium events. Rise time was defined as the time between the minimum trace timepoint within 3 s before the peak and the peak timepoint. To ensure quality GCaMP7f event data, GCaMP7f traces without any events throughout the entire recording duration were removed from further analysis. Remaining traces were manually inspected to ensure calcium events exhibited fast rise and exponential decay with minimal noise.

### 4.9. Identification of neurons activated by US

To determine whether a neuron was activated by US stimulation, we first binarized the calcium event traces using ones for the rising phase and zeros everywhere else. We then calculated the US evoked responses as the mean event rate over the 1 s period after US onset. US-evoked event rate in each neuron was then compared to the baseline shuffled distribution of the given neuron. We defined the baseline period for each neuron as the entire recording period excluding the 5 s periods following each US onset. To create the baseline shuffled distribution of event rates, we randomly selected 25 of 1-second-long periods during the baseline period during each iteration and calculated the mean calcium event rate. This procedure was iterated 1000 times with replacement. If a neuron’s US-evoked event rate is above 95th percentile of the shuffled baseline distribution, the neuron was deemed significantly activated by US. If US-evoked event rate is within 95th percentile of the baseline shuffled distribution, the neuron was deemed not modulated.

### 4.10. Pair-wise asymmetric correlation analysis

The ACC was used to estimate neural calcium event synchrony, as it represents the proportion of co-occurring calcium events between two neurons. Each neuron’s binarized calcium event trace was compared to all other neurons with the following equation:

\begin{equation*}{\text{AC}}{{\text{C}}_{A,B}} = \frac{1}{2}\left( {\frac{{\sum\limits A{{\mathop \cap \nolimits}}B}}{{\sum\limits A}} + \frac{{\sum\limits B{{\mathop \cap \nolimits}}A}}{{\sum\limits B}}} \right).\end{equation*}

To determine if US induces any long-term changes in network synchronization, we calculated the sustained ACC using the full recording duration’s calcium event traces. Short-term changes in calcium event synchrony were estimated by calculating the transient ACC over one second bins throughout the recording. For statistical comparison of ACCs between different timepoints or cellular populations, we used session averaged ACC values.

### 4.11. US-evoked GCaMP7f response characteristics

To compare population US-evoked GCaMP7f fluorescence changes, we first computed US-evoked GCaMP7f fluorescence changes in each neuron by aligning GCaMP7f trace to US onset. Evoked peak fluorescence was defined as the maximum fluorescence value within 5 s of US onset. If the peak was found after 5 s, the neuron was excluded from further response analysis.

Evoked response amplitude was calculated as the difference between evoked peak fluorescence and the minimum fluorescence value of the GCaMP7f trace between US onset and the evoked peak. Peak timing was the time from the US onset to the peak. The full-width half-maximum was defined as the time GCaMP7f fluorescence remained above 50% of the evoked response amplitude. The AUC was calculated with trapezoidal numerical integration during the 5 s post-US onset (trapz function in MATLAB).

### 4.12. Statistical analysis

We used significance level *α* = 0.05 for all statistical tests. Using quantile-quantile plots and the Shapiro–Wilkes test for normality, we determined that most of our data was not normally distributed. Accordingly, we used non-parametric statistical tests for our data analysis. When comparing relative proportions of modulated and non-modulated cells between two groups, we used Fisher’s exact test. When comparing relative proportions between three or more groups, we used a chi-square test. For comparison of medians between two groups of matched pairs, we used the Wilcoxon signed-rank test, and for comparisons between two groups of unmatched pairs we used the Wilcoxon rank-sum test. To compare medians of groups of three or more, we employed the non-parametric Kruskal–Wallis test. If a Kruskal–Wallis test was significant (*p* < 0.05), we ran a post-hoc multiple comparisons test with Dunn–Sidak corrections to test significance between groups. For comparisons of multiple repeated measures, we used a Friedman test with a post-hoc Nemenyi test.

For analyzing the influence of multiple predictor variables (i.e. PRF and cell type) on a response variable *Y* (i.e. calcium response shape characteristics), we used (GLMs, fit with function MATLAB fitglm). We used the same GLM architecture for all response variables:

\begin{equation*} Y\sim 1 + {\text{PRF}} + {\text{Cell Type}} + {\text{PRF }} \times {\text{Cell Type}}{\text{}}.\end{equation*}

Each GLM’s fit was compared to an intercept-only model with the deviance test, and we only analyzed the GLM coefficients if the deviance test was significant. As none of the GLM coefficients were significant, we did not perform post-hoc analyzes.

## Data Availability

The data cannot be made publicly available upon publication because no suitable repository exists for hosting data in this field of study. The data that support the findings of this study are available upon reasonable request from the authors.
